# Are Maternal Social Networks and Perceptions of Trust Associated with Suspected Autism Spectrum Disorder in Offspring? A Population-Based Study in Japan

**DOI:** 10.1371/journal.pone.0101359

**Published:** 2014-07-01

**Authors:** Takeo Fujiwara, Ichiro Kawachi

**Affiliations:** 1 Department of Social Medicine, National Research Institute for Child Health and Development, Setagaya-ku, Tokyo, Japan; 2 Department of Society and Behavioral Sciences, Harvard School of Public Health, Boston, Massachusetts, United States of America; Hamamatsu University School of Medicine, Japan

## Abstract

**Objective:**

To investigate the associations of maternal social networks and perceptions of trust with the prevalence of suspected autism spectrum disorders in 18-month-old offspring in Japan.

**Methods:**

Questionnaires included measurements of maternal social networks (number of relatives or friends they could call upon for assistance), maternal perceptions of trust, mutual assistance (i.e. individual measures of “cognitive social capital”), and social participation (i.e. individual measures of “structural social capital”) as well as the Modified Checklist for Autism in Toddlers to detect suspected autism spectrum disorder (ASD). These tools were mailed to all families with 18-month-old toddlers in Chiba, a city near Tokyo (N = 6061; response rate: 64%). The association between social capital or social network indicators and suspected ASD were analyzed, adjusted for covariates by logistic regression analysis.

**Results:**

Low maternal social trust was found to be significantly positively associated with suspected ASD in toddlers compared with high maternal social trust (adjusted odds ratio [OR]: 1.82, 95% confidence interval [CI]: 1.38 to 2.40); mutual aid was also significantly positively related (low vs. high: OR, 1.82, 95% CI: 1.38 to 2.40). However, maternal community participation showed U-shape association with suspected ASD of offspring. Maternal social network showed consistent inverse associations with suspected ASD of offspring, regardless of the type of social connection (e.g., relatives, neighbors, or friends living outside of their neighborhood).

**Conclusions:**

Mothers' cognitive social capital and social networks, but not structural social capital, might be associated with suspected ASD in offspring.

## Introduction

Autism, or autism spectrum disorder (ASD), is a developmental disorder typified by impaired communication and social skills [1]. Genetic factors may play a significant role in causing ASD [2], however, they do not entirely explain all cases [1]. For example, the California Autism Twins Study showed that the rates of ASD among identical twins were 77% for male and 50% for female, and for fraternal twins, 31% for male and 36% for female [3], suggesting that environmental factors may contribute to the development of ASD. Several studies investigated the association between environmental factors and ASD, including toxin exposure [4]–[8], zinc deficiency [9]–[12], or infection during pregnancy [13], [14]. However, few studies have investigated the association between social environment and ASD, or even autistic traits such as suspected ASD. Impairment of social behavior is one of the characteristics of ASD, therefore it seems plausible that characteristics of the social environment – such as the social networks surrounding the parent or the quality of social connections in the broader environment, referred to as “social capital” – might contribute to the development of ASD in the offspring. Few studies have investigated the association between social networks/social capital and autistic traits.

The term ‘social capital’ describes the resources accessed by individuals through their social networks in settings such as the community, school, or work [15]. Three key types of resources include: (a) levels of trust between individuals, (b) mutual aid, and (c) the ability to undertake coordinated, collective action. For example, a community with high social capital is one in which members frequently help each other and exchange favors. Mutual aid relies on high levels of interpersonal trust in the social network (i.e., trust that a recipient of a good deed will return the favor in the future). Therefore, social capital might have an effect on the social behaviors of children, directly or indirectly through guardians. Social capital can be assessed at both the community and individual levels: community-level social capital refers to aggregated social resources of individuals in the community, while individual-level social capital refers to each individual's perception of the quality of social interactions in the surrounding community. Recent studies have demonstrated links between oxytocin secretion and individual perceptions of social capital (i.e. individual assessments of the trustworthiness of their social connections) [15]. Moreover, oxytocin has been proposed as a treatment for autism [16]. In this context, we hypothesized that individual-level social capital as perceived by mothers and maternal social networks can be related to autistic traits in their children, even in a cross-sectional study.

Thus, the purpose of this study was to investigate the association of maternal social networks and individual perceptions of social capital with the prevalence of suspected ASD in their children in Japan.

## Methods

### Participants

The study was approved by the Ethics Committee of the National Institute for Child Health and Development. A questionnaire was mailed to all families with 18-month-old toddlers in Chiba (N = 95 000). Chiba is the capital city of Chiba Prefecture, located east of Tokyo. It is composed of six wards and has a population of around 963 000. It covers an area of 272 km^2^, making the population density around 3540 person/km^2^. Under the Japanese health care system, all mothers of newborn babies are followed up with “well baby” checkups through the local health centers. The mothers of the toddlers were requested to fill out the questionnaire and bring it to their 18-month health checkup at one of six health centers, from January to December 2011. In total, 8350 toddlers participated in the health checkup (participation rate: 87.9%), and 6106 mothers returned the questionnaires to their local health center, of which 6061 were valid for inclusion in the study (valid response rate: 63.8%).

### Measures

Maternal perceptions of social capital were assessed in both cognitive and structural domains [17]–[20]. Indicators of cognitive social capital included items inquiring about social trust and availability of mutual aid among neighbors. Social trust was assessed through a single item: “Do you think that people in your neighborhood trust each other?” Mutual aid was also assessed with a single item: “Do you think that people in your neighborhood help each other?” Response choices for both items followed a 4-point Likert-scale (“yes,” “somewhat yes,” “somewhat no,” and “no”), with each response labeled as “high,” “high-middle,” “low-middle,” and “low” trust and mutual aid, respectively [17], [18], [20].

We assessed structural social capital by asking participants about their participation in the community, which was determined by the total number of organizations in which the respondent participated. Previous studies have used this measure to demonstrate associations with health status [20], [21]. The types of organizations with which participants reported being affiliated included parenting groups, parent-teacher associations, civic organizations, consumers' cooperative societies, unions/religious groups, or other community groups [15]. Based on the distribution of responses, we classified community participation into three categories: no organizations, one organization, and more than one organizations. In addition, frequency of community participation was explored and categorized into four groups: no participation, not regularly, 1–3 times per month, and 4 or more times per month.

Social networks were assessed with the following question: “How many relatives or friends are you able to easily consult with to obtain support?” We then divided participants by the number of relatives or friends the respondents consulted with, forming three categories: 0–3, 4 or 5, and 6 or more. Further, to identify the person consulted, the following questions were asked: “Among them, how many are relatives? How many are neighbors? How many are friends who are not living near you?” Participants were again divided into three categories according to their answers.

Suspected ASD was evaluated using the Japanese version of the Modified Checklist for Autism in Toddlers (M-CHAT), which is validated [22], rated by mothers. Following the cutoff of the original M-CHAT [23], children who failed two or more of the six critical items or three or more of any of the items were considered to have suspected ASD.

The association between social capital or social network and suspected ASD was of primary interest; therefore, possible confounders identified from previous studies [24]–[27] were also evaluated through the questionnaire. These included maternal characteristics, (age, self-rated health, employment status, and marital status), family characteristics (parental education, annual household income, living with grandparents or other relatives, and number of children) and child characteristics (low birth weight, preterm, and day care attendance). Self-rated health was assessed using the following question: “How would you rate your health in general?” Response choices followed a 5-point Likert scale (excellent, very good, good, fair, or poor), but were dichotomized for analysis as: poor/fair vs. all other. Maternal and paternal educational level was categorized as junior high school graduate, high school graduate, some college/vocational school, college, or graduate school. Annual household income was assessed in increments of 2 million yen up to 10 million yen, (i.e., less than 2, 2.1–4, 4.1–6, 6.1–8, 8.1–10, 10.1–15, and 15.1 or more million yen). At current exchange rates, 1 million yen is equivalent to 10,000 USD. The median annual household income in Japan was 4.5 million yen in 2008 [28].

### Analysis

Logistic regression was used to calculate the odds ratios (OR) of suspected ASD for each social capital or social network indicator. The models adjusted for each socioeconomic status (SES) measure (i.e. parental education and income) and other potential confounders. Missing cases were treated as dummy variables to maximize statistical power. All statistical analyses were performed using the Stata MP 12, and the level of statistical significance was set at 0.05 (two-tailed).

## Results

Table 1 shows the demographic characteristics of the participants. Regarding maternal characteristics, the mean age of mothers was 32.8 years (*SD* = 4.8); 97.5% were married, 25.9% had graduated high school or less, 95.3% rated their health as “good”, and 68.9% were not working. As for household characteristics, 8.9% were living with grandparents or relatives, 53.9% had only one child, and annual income was distributed as follows: ≤4 million yen, 23.4%; 4.1–6 million yen, 32.8%; 6.1–8 million yen, 18.4%; ≥8.1 million yen, 14.0%; and missing, 11.4%. Regarding child characteristics, 7.9% had low birth weight and 26.1% attended nursery school. Suspected ASD was found in 13.5% of the sample.

**Table 1:** Characteristics of sample (N = 6061).

			N	%
Maternal characteristics	Age	<25 y	276	4.6
		25–29 y	1,220	20.1
		30–34 y	2,236	36.9
		35–39 y	1,756	29.0
		40+y	493	8.1
		Missing	80	1.3
	Marital status	Married	5,903	97.4
		Not married	151	2.5
		Missing	7	0.1
	Maternal education	HS or less	1,572	25.9
		Some college	2,670	44.1
		College+	1,658	27.4
		Missing	161	2.7
	Self-rated health	Good/very good/excellent	5,774	95.3
		Fair/poor	238	3.9
		Missing	49	0.8
	Employment status	Full-time	1,068	17.6
		Part-time	749	12.4
		Not working	4,176	68.9
		Missing	68	1.1
Household characteristics	Paternal education	HS or less	1,582	26.1
		Some college	1,210	20.0
		College+	2,978	49.1
		Missing	291	4.8
	Living with grandparents or other relatives	Yes	542	8.9
		No	5,516	91.0
		Missing	3	0.1
	Number of children	1	3,269	53.9
		2	2,135	35.2
		3+	657	10.8
	Annual household income	<400 m	1,418	23.4
		400–600 m	1,987	32.8
		600–800 m	1,117	18.4
		800 m+	851	14.0
		Missing	688	11.4
Child characteristics	Low birth weight	Yes	476	7.9
		No	5,428	89.6
		Missing	157	2.6
	Preterm birth	Yes	316	5.2
		No	5,383	88.8
		Missing	362	6.0
	Nursery school attendance	Yes	1,583	26.1
		No	4,416	72.9
		Missing	62	1.0
	Autism spectrum disorder	Suspected positive	815	13.5
		Suspected negative	5,246	86.6

The distribution of social capital and social network indicators are shown in Table 2. Social trust was found to be high for 17.9% of participants, and low for 11.5%. Similarly, high and low mutual aid was found in 18.2% and 11.5% of participants, respectively. In addition, 71.3% of participants did not participate in any community organization, while 10.4% participated in two or more community organizations. Regarding the frequency of participation, 7.9% were not regular, 14.9% participated 1–3 times per month, and 4.4% participated 4 or more times per month. The distribution of the number of relatives or friends with whom the respondents could consult easily was as follows: 0 to 3, 32.7%; 4 to 5, 30.8%; and 5 or more, 32.3%.

**Table 2:** Distribution of maternal perception of social capital and social network indicators (N = 6061).

			N	%
Cognitive social capital	Social trust	High	1,085	17.9
		High-middle	3,121	51.5
		Low-middle	957	15.8
		Low	697	11.5
		Missing	201	3.3
	Mutual aid	High	1,101	18.2
		High-middle	2,929	48.3
		Low-middle	1,149	19.0
		Low	694	11.5
		Missing	188	3.1
Structural social capital	Number of community organizations affiliated with	0	4,322	71.3
		1	1,050	17.3
		2+	608	10.4
		Missing	81	1.3
	Frequency of participation in community organization	No participation	4,320	71.3
		Not regularly	476	7.9
		1–3 times per month	900	14.9
		4+ times per month	266	4.4
		Missing	99	1.6
Social network	Number of relatives or friends who are easy to consult with	0 to 3	1,981	32.7
		4 to 5	1,864	30.8
		6+	1,959	32.3
		Missing	257	4.2
	Number of relatives who are easy to consult with	0 to 1	1,851	30.5
		2	2,197	36.3
		3+	1,763	29.1
		Missing	250	4.1
	Number of neighbors who are easy to consult with	0	1,166	19.2
		1 to 2	2,431	40.1
		3+	1,624	26.8
		Missing	840	13.9
	Number of friends outside of neighborhood who are easy to consult with	0	1,159	19.1
		1 to 2	2,494	41.2
		3+	1,318	21.8
		Missing	1,090	18.0

The ORs of suspected ASD for social capital and social network indicators are shown in Table 3. Low maternal social trust was found to be significantly positively associated with having a toddler with suspected ASD in comparison with high maternal social trust (crude model: odds ratio [OR]: 1.97, 95% confidence interval [CI]: 1.51 to 2,57). Results were similar even after adjustment of maternal, household, and infant characteristics (adjusted OR: 1.82, 95% CI: 1.38 to 2.40). The gradient effect of social trust on having a toddler with suspected ASD was also found to be significant (p for trend <0.001). Similarly, mutual aid was also significantly associated with having a toddler with suspected ASD (low vs. high: adjusted OR, 1.82, 95% CI: 1.38 to 2.40) with a significant gradient effect (p for trend <0.001). By contrast, community participation had a marginally significant association with having a toddler with suspected ASD in the crude model (p for trend  = 0.049), which became non-significant in the adjusted model (p for trend  = 0.094). Interestingly, frequency of community participation showed a U-shape association with having suspected ASD offspring. It was found that mothers who participated in community activities 1–3 times per month were 25% less likely to have a toddler with suspected ASD (adjusted OR: 0.75, 95% CI: 0.60 to 0.95) in comparison with the no-community-participation group. On the other hand, participating 4 or more times per month was not associated with having a toddler with suspected ASD (adjusted OR: 0.96, 95% CI: 0.66 to 1.39).

**Table 3:** Odds ratio of maternal perception of social capital and social network indicators for offspring's suspected ASD.

			Crude		Adjusted	
			OR	95% CI	OR	95% CI
Cognitive social capital	Social trust	High	Reference		Reference	
		High-middle	1.10	0.88 to 1.36	1.09	0.87 to 1.36
		Low-middle	**1.60**	**1.23 to 2.06**	**1.54**	**1.19 to 2.00**
		Low	**1.97**	**1.51 to 2,57**	**1.82**	**1.38 to 2.40**
		P for trend	**<0.001**		**<0.001**	
	Mutual aid	High	Reference		Reference	
		High-middle	1.12	0.90 to 1.40	1.11	0.89 to 1.39
		Low-middle	**1.40**	**1.09 to 1.80**	**1.35**	**1.05 to 1.74**
		Low	**2.24**	**1.72 to 2.91**	1.82	1.38 to 2.40
		P for trend	**<0.001**		**<0.001**	
Structural social capital	Number of community organizations affiliated with	0	Reference		Reference	
		1	0.89	0.73 to 1.09	0.91	0.74 to 1.11
		2+	0.79	0.60 to 1.03	0.81	0.62 to 1.07
		P for trend	**0.049**		0.094	
	Frequency of participation in community organization	No participation	Reference		Reference	
		Not regularly	1.05	0.80 to 1.37	1.12	0.85 to 1.47
		1–3 times per month	**0.75**	**0.60 to 0.94**	**0.75**	**0.60 to 0.95**
		4+ times per month	0.97	0.67 to 1.39	0.96	0.66 to 1.39
		P for trend	0.072		0.096	
Social network	Number of relatives or friends to consult with	0 to 3	Reference		Reference	
		4 to 5	**0.66**	**0.55 to 0.79**	**0.66**	**0.55 to 0.79**
		6+	**0.55**	**0.45 to 0.66**	**0.56**	**0.46 to 0.67**
		P for trend	**<0.001**		**<0.001**	
	Number of relatives to consult with	0 to 1	Reference		Reference	
		2	**0.65**	**0.55 to 0.78**	**0.65**	**0.55 to 0.78**
		3+	**0.58**	**0.48 to 0.71**	**0.58**	**0.48 to 0.71**
		P for trend	**<0.001**		**<0.001**	
	Number of neighbors to consult with	0	reference		reference	
		1 to 2	**0.68**	**0.56 to 0.82**	**0.71**	**0.58 to 0.86**
		3+	**0.57**	**0.46 to 0.71**	**0.61**	**0.49 to 0.77**
		P for trend	**<0.001**		**<0.001**	
						
	Number of friends outside of neighborhood to consult with	0	reference		reference	
		1 to 2	**0.75**	**0.62 to 0.92**	**0.74**	**0.60 to 0.90**
		3+	**0.74**	**0.59 to 0.93**	**0.71**	**0.57 to 0.90**
		P for trend	**0.009**		**0.007**	

Maternal social network showed consistent inverse associations with having offspring with suspected ASD, regardless of relatives, neighbors, or friends living outside of their neighborhood. For example, participants who had 4 to 5 relatives or friends to consult with were 34% less likely to have a toddler with suspected ASD (adjusted OR: 0.66, 95% CI: 0.55 to 0.79). Similarly, participants who had 6 or more relatives or friends to consult with were 44% less likely to have a toddler with suspected ASD (adjusted OR: 0.56, 95% CI: 0.46 to 0.67), and the gradient was statistically significant (p for trend <0.001).

## Discussion

The current study showed that a mother with low social trust is more likely to have a toddler with suspected ASD in comparison with a mother with high social trust. The results were similar in the case of mutual aid. However, structural social capital (i.e., maternal membership of community organizations) showed no linear association with having a child with suspected ASD, although a U-shaped association was found for frequency of community participation. Maternal social network showed consistent inverse association with having a child with suspected ASD, regardless of types of consultants, relatives, neighbors, or friends living outside their neighborhood.

To the best of our knowledge, this is the first study to show an association between social capital and prevalence of suspected ASD. However, because this study was a cross-sectional study, the causality of social capital on having a toddler with suspected ASD could not be determined—in other words, we could not explore whether having offspring with autistic traits directly induces low social capital or low social networks of parents. Moreover, previous studies including a study from Japan reported that parents who had children with ASD were less likely to have poor social relationships [29], [30]. Nonetheless, the possibility of social causation remains. Previous studies reported associations of toxin exposure [4]–[8], zinc deficiency [9]–[12], and infection during pregnancy [13], [14] with ASD, suggesting that mothers living in disadvantaged communities might be more likely to be exposed to toxic pollutants, or zinc deficiency due to poor access to food. Because low social capital communities are more likely to be disadvantaged [31], exposure to these risk factors might contribute to the association between maternal social capital and ASD. Possibly, characteristics of social environment – viz., the strength of social networks or local social capital – could enhance the quality of interaction between infants and caregivers [32], which might have an effect on the development of autistic traits [33].

Alternatively, the association found in this study might be confounded by shared genetic predisposition between mothers and infants. Mothers with a predisposition for autistic traits may be more likely to end up in less socially interactive community settings, such as rented housing [34], in which social capital is low. Due to the heritable component of ASD [2], mothers with an autistic predisposition are more likely to have offspring with autistic traits. Further study is needed to confirm the association between social capital and toddlers with suspected ASD, with adjustment for maternal autistic traits. Nonetheless, the current finding is significant because low social capital can be a marker to identify communities with a higher proportion of toddlers with suspected ASD, which might be useful from a needs assessment standpoint.

We found a U-shape association between frequency of community participation and having suspected ASD offspring. This finding is important because mothers who are frequent participants in community organizations are less likely to have autistic traits. However, it is also important to consider that maternal autistic traits are not necessarily linearly associated with having offspring with autistic traits. In addition, this finding suggests that appropriate frequency of community participation (1–3 times per month) for mothers is important for the social development of children, in comparison with a lack of community participation or too much community participation. A previous study reported that participation in education or support groups is beneficial for self-esteem, parenting skills, and communication with children [35]. Thus, frequency of community participation can be considered as a factor affecting the social development of offspring indirectly by affecting the parents' social and parenting skills. Alternatively, mothers who have a child with suspected ASD may be more likely to participate in several community programs or attend parenting support groups for children with developmental problems. Further research is needed to elucidate the causation of the association between maternal community participation and autistic traits of offspring.

We investigated different sources of maternal network support, including relatives, neighbors, and friends not living in the neighborhood, and found that all three types were consistently inversely associated with having a child with suspected ASD. This suggests that mothers who have a child with suspected ASD are less likely to have high social skills, probably due to maternal autistic traits. A previous study reported that the physical distance of a friend affects people's happiness [36]. By contrast, it was found that in Chiba city, physical distance did not matter in terms of the prevalence of suspected ASD. This might be due to technology such as mobile phones, cars, or the internet, which may help individuals to stay in touch with social contacts irrespective of distance.

In addition to the previously mentioned points, other limitations of this study need to be addressed. First, as the M-CHAT screening scale was used to detect suspected ASD, misclassification of suspected ASD (i.e., false positive) might have resulted in underestimation of the association of social capital or social network with having a child with suspected ASD. However, as the prevalence of suspected ASD in our study (13.5%) was equivalent to the prevalence of suspected ASD among 18-month-old toddlers who were identified for follow-up in a previous study (i.e. 14.3%) [37], the assessment of suspected ASD is considered acceptable. Second, information on paternal characteristics such as age or occupation was not assessed, although previous studies reported that they might be independently associated with ASD [38], [39]. However, paternal age could be correlated with maternal age, and we found no association with maternal age and suspected ASD in offspring (data not shown). In addition, although occupation was not considered, paternal education was adjusted for in the analysis.

In conclusion, maternal cognitive social capital and social networks, but not structural social capital, was associated with having a child with suspected ASD. Maternal social network was also associated with having a child with suspected ASD, regardless of types of social contacts. Further research is needed to elucidate the association between maternal cognitive social capital and the development of autistic traits in offspring.
