# Correction: Are Maternal Social Networks and Perceptions of Trust Associated with Suspected Autism Spectrum Disorder in Offspring? A Population-Based Study in Japan

**DOI:** 10.1371/journal.pone.0104332

**Published:** 2014-07-28

**Authors:** The PLOS ONE Staff

There is an error in the odds ratio for mutual aid in the Abstract, Results section, and Table 3.

**Table 3:** Odds ratio of maternal perception of social capital and social network indicators for offspring's suspected ASD

			Crude		Adjusted	
			OR	95% CI	OR	95% CI
Cognitive social capital	Social trust	High	Reference		Reference	
		High-middle	1.10	0.88 to 1.36	1.09	0.87 to 1.36
		Low-middle	**1.60**	**1.23 to 2.06**	**1.54**	**1.19 to 2.00**
		Low	**1.97**	**1.51 to 2,57**	**1.82**	**1.38 to 2.40**
		P for trend	**<0.001**		**<0.001**	
	Mutual aid	High	Reference		Reference	
		High-middle	1.12	0.90 to 1.40	1.11	0.89 to 1.39
		Low-middle	**1.40**	**1.09 to 1.80**	**1.35**	**1.05 to 1.74**
		Low	**2.24**	**1.72 to 2.91**	2.08	1.59-2.73
		P for trend	**<0.001**		**<0.001**	
Structural social capital	Number of community organizations affiliated with	0	Reference		Reference	
		1	0.89	0.73 to 1.09	0.91	0.74 to 1.11
		2+	0.79	0.60 to 1.03	0.81	0.62 to 1.07
		P for trend	**0.049**		0.094	
	Frequency of participation in community organization	No participation	Reference		Reference	
		Not regularly	1.05	0.80 to 1.37	1.12	0.85 to 1.47
		1-3 times per month	**0.75**	**0.60 to 0.94**	**0.75**	**0.60 to 0.95**
		4+ times per month	0.97	0.67 to 1.39	0.96	0.66 to 1.39
		P for trend	0.072		0.096	
Social network	Number of relatives or friends to consult with	0 to 3	Reference		Reference	
		4 to 5	**0.66**	**0.55 to 0.79**	**0.66**	**0.55 to 0.79**
		6+	**0.55**	**0.45 to 0.66**	**0.56**	**0.46 to 0.67**
		P for trend	**<0.001**		**<0.001**	
	Number of relatives to consult with	0 to 1	Reference		Reference	
		2	**0.65**	**0.55 to 0.78**	**0.65**	**0.55 to 0.78**
		3+	**0.58**	**0.48 to 0.71**	**0.58**	**0.48 to 0.71**
		P for trend	**<0.001**		**<0.001**	
	Number of neighbors to consult with	0	reference		reference	
		1 to 2	**0.68**	**0.56 to 0.82**	**0.71**	**0.58 to 0.86**
		3+	**0.57**	**0.46 to 0.71**	**0.61**	**0.49 to 0.77**
		P for trend	**<0.001**		**<0.001**	
	Number of friends outside of neighborhood to consult with	0	reference		reference	
		1 to 2	**0.75**	**0.62 to 0.92**	**0.74**	**0.60 to 0.90**
		3+	**0.74**	**0.59 to 0.93**	**0.71**	**0.57 to 0.90**
		P for trend	**0.009**		**0.007**	

In both the Results section of the Abstract and the third paragraph of the Results section, the odds ratio for mutual aid should read “low vs. high: OR, 2.08, 95% CI: 1.59 to 2.73.”

The odds ratio in Table 3 is incorrect. Please see the corrected version of the table below.
